# Bayesian Network Analysis for Prediction of Unplanned Hospital Readmissions of Cancer Patients with Breakthrough Cancer Pain and Complex Care Needs

**DOI:** 10.3390/healthcare10101853

**Published:** 2022-09-23

**Authors:** Marco Cascella, Emanuela Racca, Anna Nappi, Sergio Coluccia, Sabatino Maione, Livio Luongo, Francesca Guida, Antonio Avallone, Arturo Cuomo

**Affiliations:** 1 Division of Anesthesia and Pain Medicine, Istituto Nazionale Tumori, IRCCS Fondazione G. Pascale, 80131 Napoli, Italy; 2 DIETI, University Federico II, 80100 Naples, Italy; 3 Clinical Sperimental Abdominal Oncology Unit, Istituto Nazionale Tumori, IRCCS Fondazione G. Pascale, 80131 Napoli, Italy; 4 Epidemiology and Biostatistics Unit, Istituto Nazionale Tumori, IRCCS Fondazione G. Pascale, 80131 Napoli, Italy; 5 Department of Experimental Medicine, Division of Pharmacology, University of Campania Naples, 80138 Naples, Italy; 6 Neuromed, IRCCS Pozzilli, 86077 Pozzilli, Italy

**Keywords:** Bayesian analysis, hospitalization, quality of life, predictive models, cancer pain, breakthrough cancer pain

## Abstract

Background: Unplanned hospital readmissions (HRAs) are very common in cancer patients. These events can potentially impair the patients’ health-related quality of life and increase cancer care costs. In this study, data-driven prediction models were developed for identifying patients at a higher risk for HRA. Methods: A large dataset on cancer pain and additional data from clinical registries were used for conducting a Bayesian network analysis. A cohort of gastrointestinal cancer patients was selected. Logical and clinical relationships were a priori established to define and associate the considered variables including cancer type, body mass index (BMI), bone metastasis, serum albumin, nutritional support, breakthrough cancer pain (BTcP), and radiotherapy. Results: The best model (Bayesian Information Criterion) demonstrated that, in the investigated setting, unplanned HRAs are directly related to nutritional support (*p* = 0.05) and radiotherapy. On the contrary, BTcP did not significantly affect HRAs. Nevertheless, the correlation between variables showed that when BMI ≥ 25 kg/m^2^, the spontaneous BTcP is more predictive for HRAs. Conclusions: Whilst not without limitations, a Bayesian model, combined with a careful selection of clinical variables, can represent a valid strategy for predicting unexpected HRA events in cancer patients. These findings could be useful for calibrating care interventions and implementing processes of resource allocation.

## 1. Introduction

Cancer patients are at a higher risk of unplanned hospital readmissions (HRAs). The complex nature of the cancer disease and high-intensity care needs are the main causes [1]. Diagnostic and/or therapeutic procedures including chemotherapy, radiotherapy, and surgical interventions are frequent reasons for HRAs. Moreover, symptoms and complications of the oncologic disease and potential comorbidities can require acute hospital care. Gastrointestinal (GI) obstruction, dyspnea, and altered mental status are the leading clinical conditions [2,3].

Among cancer types, GI cancers are particularly common [4,5,6]. In these patients, chemotherapy and its related toxicity can increase the risk for HRA [4]. Other potential risk factors include receipt of radiation therapy, advanced disease stages, and comorbidities such as diabetes or chronic obstructive pulmonary disease [4,7,8]. Furthermore, a recent investigation found that other demographic and clinical conditions are significantly associated with increased odds of hospitalization. These factors include female gender, diagnosis of gastric/esophageal cancer, polypharmacy (≥5 daily medications), decreased hearing, and patient-reported cardiac comorbidity (history of heart disease), as well as low serum albumin (<3.5 g/dL) [9].

Cancer pain occurs in 20–30% of cases during the initial stages and in up to 75% of patients with advanced disease. The prevalence of cancer pain at any stage of the disease is over 50%. Concerning pain intensity, moderate-to-severe pain can affect up to 40% of all patients. Moreover, this symptom strongly affects the patient’s health-related quality of life (HRQoL) and daily activities throughout the disease course [10]. In the context of cancer pain phenomena, breakthrough cancer pain (BTcP) represents an unexpected worsening of pain despite adequate control of the background pain through opioid therapy [11]. This type of pain can affect up to 70% of cancer patients and is associated with significant morbidity and negative outcomes [10,11,12]. Remarkably, in cancer patients, pain issues seem to be the most frequent reason for hospitalization [13].

Hospitalization largely induces a rise in cancer-related health care spending. Usually, hospital treatments for cancer last longer and cost more than those for other clinical conditions [14]. Since the literature suggested that, in advanced cancer patients, HRA is largely aimed at symptom control [3], identifying subgroups of patients at increased risk could be a key strategy for reducing this phenomenon. Consequently, decreasing potentially avoidable hospitalizations is a promising target for improving the patients’ HRQoL and reducing cancer care costs [14]. Furthermore, having a clear idea of the phenomenon can stimulate the use of calibrated approaches, such as telemedicine strategies [15,16].

There is an increased interest in Bayesian statistical inference in public health and medical research. Many applications are in the field of cancer prediction and prognosis [17], but several pieces of research have been conducted in palliative care [18] as well as in other clinical settings and scenarios [19,20,21]. Interestingly, in large cohort of inpatients (*n* = 198,972), Roth et al. [22] investigated HRAs from all causes using Bayesian data-driven analytical methods. A similar approach was recently implemented for evaluating readmissions in patients with peripheral vasculopathy [23]. Bayesian methods provide mathematical tools to rationally update subjective beliefs in the light of new data or evidence. This contrasts with classical or frequentist statistical inference, which presumes that the probabilities are the frequency of particular random events occurring in a long series of repeated trials. Thus, the peculiarity of a Bayesian network is to recalculate the probabilities of a target event (a posteriori) after placing conditions on other (“causing”) events in order to measure how much these values vary. The state of a priori knowledge is updated to provide the state of knowledge after (a posteriori) the study. In other words, a Bayesian approach allows a robust estimate based on the data but also exploits the information (e.g., correlations) on considered elements. The assumption is that although models must offer predictive results with excellent performance, often there is the need to provide an accurate estimate of the uncertainty of the prediction [20]. Thus, these Bayesian approaches are also increasingly used for predictive analysis in machine learning and artificial intelligence [17,24].

On these premises, this study was aimed at the development of data-driven prediction models for identifying those patients at a higher risk for unplanned HRA. A Bayesian network approach was implemented. Results could be useful for calibrating care interventions and for better resource allocation.

## 2. Materials and Methods

### 2.1. Data Sources

This study is based on the dataset of the Italian Oncologic Pain Multicentric Survey (IOPS-MS). This investigation was carried out on a large number of patients (*n* = 4016) for dissecting different cancer pain phenomena and providing information on BTcP [25].

From the IOPS-MS original dataset, data of patients enrolled and treated at the Abdominal Oncology service of the Istituto Nazionale Tumori-Fondazione Pascale from January 2014 to April 2015 were extrapolated. Clinical data and biochemical tests were collected from the patient’s digital medical records while information about background pain, BTcP, and analgesic therapy was collected from the patient’s Case Report Form. All data were reported on an Excel file and then registered on Zenodo [26]. Included patients were 18 years of age or older, had a confirmed histological diagnosis of GI malignant neoplasm, and were on active chemotherapy with first or subsequent lines of chemotherapy. For each patient, demographic and clinical data were collected (Table 1) for evaluating the correlations between different variables.

The IOPS study was conducted by following the Declaration of Helsinki’s ethical principle. Approval from the Institutional Medical Ethical Committee (protocol 32/14 OSS) of the Istituto Nazionale Tumori-Fondazione Pascale, Naples was obtained, and patients signed informed consent before enrolling in the study.

### 2.2. Data Preprocessing and Model Building

The model adopted was a multinomial Bayesian network. It is a cause–effect model structure built on a DAG that is an easy visualization of the direct causal relations between features: such sorts of direct dependencies are drawn as directed arches. Variables that are not linked by an arc are treated as conditionally independent. Thus, given three elements A, B, and C, A involves B which involves C, once data are given; knowing A does not influence the probability of C given B, P(C|A,B) = P(C|B). Such a recording of that statistical problem allows for reducing the parameters of the joint distribution of the whole A, B, and C probabilistic structure of the set.

As in parametric methods, Bayesian networks can be submitted to parameters’ reduction and model selection. The structure of the DAG can be chosen by considering any plausible association/dependence between the causing and the caused variables and a likelihood formulation can be obtained for such a model; it is possible to preserve the most significant features by assessing the usual tests for evaluating the local significance of the single parameters. It is also possible to measure the global performance of it by goodness-of-fit tests. Exact and approximate inference can be assessed to analyze the features’ relations of interest, which are reformulated as posterior probabilities. Direct causal relations, summarized by arches, act to reformulate the theory of conditional/conditioned events so that they can easily draw the given probabilistic schema for the training sample. Consequently, the actual revelation of individuals, in the discrete case their probabilities of being in a class (specified by parameters), is synthesized in a manageable way based on these relations.

The methods can be summarized in the following steps:

1. Data preprocessing and discretization using cut points of clinical interest (e.g., serum albumin values and the number of hospital accesses).
2. Selection of a subset of variables.
3. Evaluation of significant associations (Pearson’s Chi-square test).
4. Development of white- and blacklists relating to arches (variables associations), according to logic and clinical criteria.
5. Design of a “knowledge-based” model containing the associations (causality, therefore directed) between variables as a causal directed acyclic graph (DAG).
6. Significance analysis of the arches (relationships).
7. Estimation of the Bayesian network model according to goodness indicators for the Bayesian Information Criteria (BIC) (also termed as Schwarz Criterion). It is linked to the likelihood of the model regarding the estimated parameters and contains associations between variables. Theoretical models are validated according to the BIC minimization or other indicators (e.g., Akaike Information Criteria, AIC, Bayesian Dirichlet Equivalent) [27].
8. Choice of the BIC model due to observation penalization balance (its reliability decreases as the number of observations increases) and implementation of the Bayesian network for exact inference [28]. See the following formula where *k* indicates the number of parameters estimated by the model; *n* is the number of observations; *θ* is the set of parameters; and *L*(*θ*) represents the maximized value of the likelihood function of the model:

$$BIC:\;k\cdot ln\left(n\right)-2\cdot ln\left[L\left(\theta \right)\right]\;$$

9. Causal inference on the sample for main clinical interest queries.

Data preprocessing and model building were performed using the *R* software, version 4.1.3 (R Core Teams, R Foundation for Statistical Computing, Vienna, Austria). The toolkit included bnlearn (Scutari, Denis), gRbase, and gRain for model implementation. The suites ggplot2 and Rgraphviz were adopted for visualization. The Chi-square test was used for categorical variables.

## 3. Results

**Figure 1:**
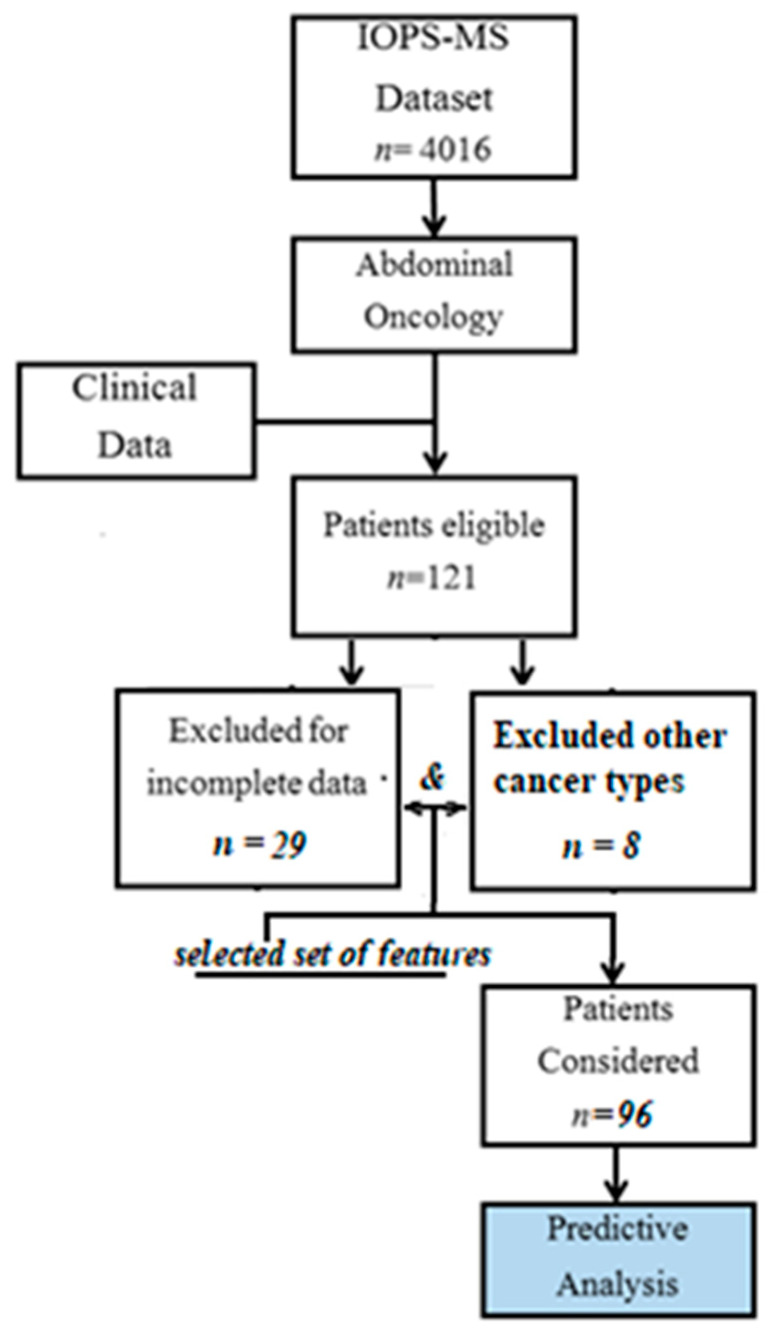
Study Flowchart.

From the original IOPS dataset (*n* = 4016), 121 eligible patients were extrapolated, and clinical data were retrieved. Twenty-nine patients were excluded for incomplete data; patients with different neoplasms from esophageal, gastro, colorectal, pancreas, gallbladder, and biliary tract (*n* = 8) were also excluded. Finally, data from 96 patients were considered for the predictive analysis (Figure 1).

Demographic and clinical data are reported in Table 2.

Although all the variables concur to determine a certain effect on the main outcome (in this investigation, HRAs), adopting the whole set is disadvantageous since a more composed number of variables can impair model generalization. Therefore, the analysis was focused on some clinical phenomena such as pain features and nutrition. Other variables were excluded for inconsistent data. For example, since only two individuals had no metastases, the adopted variable was “bone metastasis” (more consistent data). Moreover, the variable chemotherapy line was discarded due to exceeding missing data. Cancer patients’ age was almost high (Table 2) and no strong information came from such variable. Several simulations indicated that background pain features did not offer additional elements to the analysis, not even by manipulating subsets (e.g., combining the different types of pain). In the final analysis, the following were considered:

▪ Cancer type
▪ Body mass index (BMI)
▪ Bone metastasis (MTX)
▪ Albumin (ALB)
▪ Nutritional support (NUTR)
▪ Breakthrough cancer pain (BTcP)
▪ Radiotherapy (RADIO)
▪ Hospital readmission (HRA)

In the subsequent processing step, logical and clinical relationships were a priori established, and white- and blacklists were obtained:

- Whitelist. Certain relationships must be necessarily valid. Even if the relationship is not certain, it must be reported in the graph model, because, validated by the theory:
  ○ About BTcP, higher BMI can be linked to greater pain severity [29].
  ○ In some types of cancer (prostate cancer, breast cancer, and others), bone metastases are more common; in others, the metabolic effort is more evident (e.g., pancreatic cancer). Thus, the correlations of cancer type and bone metastases with HRAs were included in the whitelist.
  ○ Bone metastases induce BTcP, as well as palliative radiotherapy and nutritional needs [30].
  ○ There is a clinical correlation between albumin values and nutritional support.
- Blacklist. Impossible relationships.
  ○ A tumor (“cancer”) cannot be caused by the other considered variables.
  ○ BTcP cannot be caused by albumin and nutritional support.
  ○ Albumin and nutritional support cannot cause bone metastasis and nutritional needs.

The lists were used for the association analysis. Table 3 shows the analysis of the covariates according to the obtained dataset. Results are subsequently implemented for the construction of the Bayesian model. Cause and effect relationships (model arches) were assigned between variables. These relationships were established according to a logical relationship and clinical criteria.

Associations were found between:

- BMI and BTcP.
- Cancer type and bone metastasis, nutritional support, and HRAs.
- Bone metastasis and cancer type, nutritional support, BTcP, and radiotherapy.
- Albumin with nutritional support. Motivation: clinical relationships.
- Nutritional support and cancer type, bone metastasis, albumin, radiotherapy, and HRAs.
- BTcP with BMI and bone metastasis.
- Radiotherapy with bone metastasis and nutritional support.
- HRAs with cancer type and nutritional support.

**Figure 2:**
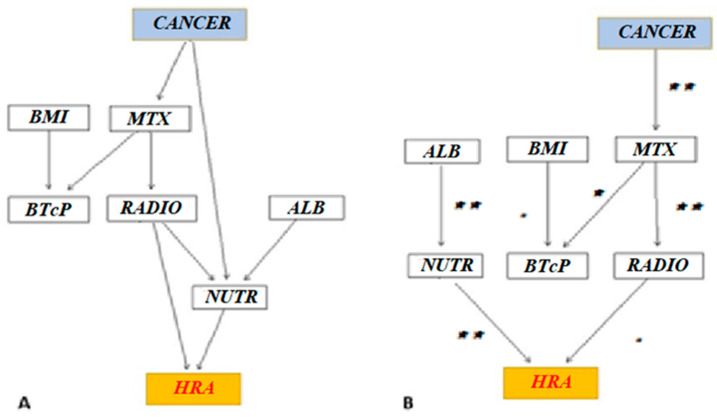
Bayesian network analysis. Hypothesized model (knowledge-based DAG) (**A**) and automatically learned model with imposed constraints (e.g., cancer and MTX) (BIC-based DAG) (**B**). The BIC-based DAG is obtained through logical and clinical associations and by selecting the model (DAG) with the best (i.e., lowest) BIC. Legend: DAG: directed acyclic graphic; BIC: Bayesian Information Criterion. Abbreviations: BMI, body mass index; MTX, metastasis; ALB, albumin; NUTR, nutritional support; BTcP, breakthrough cancer pain; RADIO, radiotherapy; HRA, hospital readmission. (** = *p* < 0.05; * = *p* < 0.10; • = Not Significant).

On these bases, structures for Bayesian networks (DAGs) were built. According to the hypothesized model (knowledge-based DAG model), HRAs were directly linked to radiotherapy and nutritional support. On the contrary, HRAs were conditionally independent of MTX and albumin values, respectively. The unplanned accesses were not directly associated with BTcP and BMI values (Figure 2A).

Later, based on the fixed links of the white- and blacklists, the best graph model (Bayesian Information Criterion, BIC) was obtained (BIC-based DAG).

Table 4 summarizes the main indicators of the two models of Bayesian networks (namely knowledge-based and BIC-based).

The formula of the BIC-based model was:

$$BIC\;based\;model:\;P\left(BMI\right)P\left(ALB\right)\cdot P\left(CANCER\right)\cdot P(MTX|CANCER)\cdot P(NUTR|ALB)\;\;\;\;\;\;\;\;\;\;\cdot P(BTcP|MTX,BMI)\cdot P(RADIO|MTX)\cdot P(HRA|NUTR,RADIO)$$

A Chi-square test on arches’ links showed that radiotherapy and cancer type were not linked to nutrition (and albumin). HRAs were directly related to nutritional support (*p* = 0.05) and radiotherapy, although the link between HRA and radiotherapy was not significant (*p* = 0.6, whitelist). Finally, BTcP did not significantly affect HRAs (Figure 2B).

As Scutari and Denis [31] showed, the Bayesian network was trained to obtain conditional probabilities and causal inference investigations.

**Figure 3:**
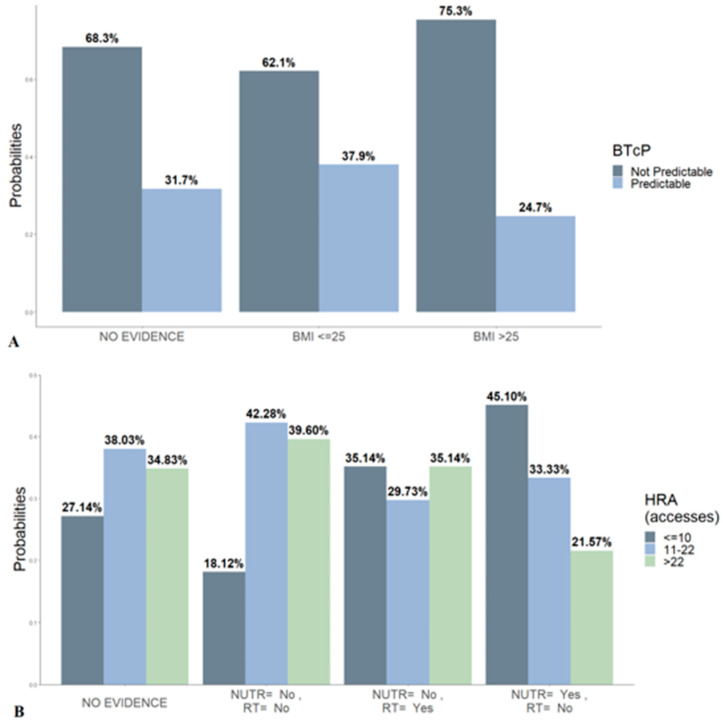
Conditional probabilities for BTcP (**A**) and hospital readmission (**B**). Bayesian inference on nutritional support and radiotherapy. Marginal values are overall data without variables conditioning. Abbreviations: BTcP, breakthrough cancer pain; RT, radiotherapy; NUTR, nutritional support; HRA, hospital readmission.

Two conditional probability queries were given for exact inference. Regarding BTCP type (Figure 3A), the estimated percentages of predictable and non-predictable BTcP subtypes were 31.7% and 68.3%, respectively. There was no evidence of the relationship with the BMI status. When the evidence on BMI was imposed, the non-predictable BTcP showed a 13-percentage point increase with respect to the predictable type, if BMI ≥ 25 kg/m^2^.

With respect to the number of accesses, the model predicted that just over a quarter of patients (27.14%) can undergo fewer than 10 HRAs, whereas 38% can undergo between 11 and 22 accesses, and 34.8% can undergo more than 22. Nevertheless, considering the features directly associated with the HRAs (i.e., RADIO and NUTR), a Bayesian inference was performed to recalculate the posterior probability of HRAs. The association of the two variables does not alter the probability of access. Cancer patients who do not receive nutritional support and radiotherapy are more likely to increase HRAs. Those who receive nutritional support, but not radiation therapy, are less likely to return to the hospital (≤10 HRAs = 45.1%). Data are inconsistent for the inference of the option nutrition plus radiotherapy.

## 4. Discussion

In cancer patients, multiple causes can induce unscheduled access to the hospital [14]. Since reducing acute care is mandatory, a predictive model can help in the management of this vulnerable population. In this complex scenario, the question to be answered is what are the variables that, also indirectly, can affect the outcome (i.e., unplanned HRAs). For this aim, a Bayesian network approach can be useful as it agrees with a logic structure from the data and allows optimal prediction combined with a useful causal inference process. As we showed, this approach can enable the learning of reliable structures in the context of causal relations [27]. Among the Bayesian networks, the BIC-based estimated model can develop certain relationships that are a priori established by the clinician. This model is more robust because, due to reduced relationships, the analysis is less affected by outliers. In other words, it is a criterion for model selection among a finite set of options. Consequently, strategies like BIC minimization can be generalized and capture more varied patterns [28,32].

In patients with GI malignancy, low albumin levels and the need for nutritional support are the main variables responsible for returning to the hospital (Figure 2B). This finding is consistent with what was previously underlined in other studies. In this clinical setting, it was shown that reduced serum albumin values may be related to increased care needs and significatively impact patients’ survival [33]. Additionally, the inference on the two variables that the model indicated as directly related to the outcome suggested that poor attention to nutritional support and radiotherapy-related issues or palliative radiotherapy requirements (e.g., for hemostasis of cancer bleeding) increased the rate of hospital readmissions. Regarding toxicities, Tey et al. [34] showed that, in GI cancer, severe toxicities develop in up to 15% of patients treated with radiotherapy alone and in approximately a quarter of patients treated with chemoradiotherapy. Notably, in this setting, the optimal dose fractionation regimen for symptom palliation should be better investigated. 

Many aspects of the BTcP phenomenon must be necessarily better clarified. BTcP is an umbrella term that encompasses a heterogeneous group of clinical manifestations. Research gaps mostly concern its pathophysiology, potential triggering factors, and clinical manifestations [35]. In our analysis, a significant association (*p* < 0.10) was found between the presence of bone metastases and BTcP. This finding confirms our expectations because bone secondaries represent the main cause of predictable BTcP [25,36]. Moreover, the analysis showed that spontaneous (or non-predictable) BTcP is also related to metastasis. In addition, a higher risk of unpredictable BTcP was calculated for elevated BMI values. This finding is in contrast with results from a recent large-sized observational study on the topic that reported no significant difference in BTcP incidence among different BMI values [37]. On the contrary, the variable BTcP was not probabilistically linked to an increased rate of unplanned hospitalizations. It is conceivable that the management of BTcP can take place effectively through a careful follow-up program in the clinics. However, many episodes of BTcP still remain not recognized and lead to a deleterious impact on the HRQoL [10]. Despite further studies on BTcP being mandatory, our results can add useful elements to the understanding of a clinical phenomenon that has not yet been exhaustively investigated [38].

The developed model demonstrates a non-dependent relationship between cancer type and HRAs. Therefore, no significant variations can be expected in the rate of unplanned accesses (probabilities) based on the oncological pathology.

From a perspective, the results of the Bayesian network analysis suggest that calibrated programs are needed to identify cancer patients at risk of hospitalization. For example, enhancement of nutritional support in this setting is required and outpatient treatment modalities need to be strengthened. Furthermore, strategies of early palliative care are required for avoiding unnecessary and expensive long hospital stays. Finally, alternative modalities such as the use of telemedicine require effective implementation.

### 4.1. Limitations

The main limitation of the study is the sample size. This issue limited the ability to evaluate the quality of the network. We focused on the training of the BIC-based model and showed the sample behavior as a causal descriptive inference only in the whole sample. Consequently, training and testing of the model were not possible. Nevertheless, as underlined in the Materials and Methods section, our aim was to describe oncological patients with abdominal cancer sites, categorized by similitude in cancer type and clinical features. Although the IOPS study offered information mainly on pain, data concerning the other variables (e.g., clinical data and biochemical tests) collected from the patient’s digital medical records were not complete in the clinical records. Unfortunately, only a small number of samples met the requirements.

The choice of categorizing continuous values is another limitation. This approach can imply losing information. However, by carrying out the association tests (both Chi-square and mutual information from a conditional Gaussian feature for mixed variables) the relationships between features are exactly the same, 95%.

The development of Bayesian models involves the knowledge of relationships not refuted by the data. Therefore, the study design must be rigorously planned. However, a close collaboration between clinicians and analysts can enhance the results.

Another major limitation is the need for a priori variable manipulation. This technical step involves the absence of a well-defined objective criterion. In our analysis, several variables such as the type of background pain (nociceptive, neuropathic, or mixed), cancer stage, performance status, and the need for surgery were excluded. Although the analysis of the probabilities of HRAs related to the various therapeutic options for cancer pain (e.g., opioids) was one of the objectives of the study, these features were discarded because they could not be inserted into the model (absence of logical or mathematical correlation during the simulations).

Another limitation is the lack of a stratification of the underlying causes of hospitalization. In this regard, Whitney et al. [14] demonstrated that infection and complications of a medical device or care are the main causes of unplanned HRAs. Although a cause analysis would have given more weight to our results, the datasets used did not report the reasons for acute hospitalizations. Based on our findings, it may be possible to define prospective studies or establish criteria for a detailed retrospective data collection.

### 4.2. Clinical and Research Perspectives

The perspectives that arise from the results of the analysis concern possible implementations of care strategies and research perspectives. In particular, in terms of clinical applications, the knowledge of the factors involved in the need for unscheduled access can stimulate the design of personalized paths. At the same time, this step presupposes a review of care processes with a better allocation of resources. For example, personalized treatments can also include remote approaches, taking advantage of the various possibilities offered by telemedicine [39].

Concerning research perspectives, with larger samples, artificial intelligence methods could be built to validate predictive models for improving decision-making processes and the efficiency and quality of healthcare services. It represents a unique opportunity to enhance patient care. Remarkably, the possibility of being able to operate on big data must stimulate research. Predictive investigations based on multisource datasets are needed and several datasets collected for cancer investigations can be adopted for this aim. Moreover, since a high quality of data must be guaranteed, close collaboration between clinicians, data managers, and IT scientists is necessary. Finally, prospective clinical investigations are needed to verify the effectiveness of care programs based on findings offered by Bayesian statistics and other mathematic approaches.

## 5. Conclusions

Since unplanned HRAs can negatively impact patients’ HRQoL and increase healthcare costs, it is essential to evaluate the underlying factors. Bayesian network analysis can represent a valid strategy. Nevertheless, a careful selection of clinical variables is required and close collaboration between clinicians and analysts is mandatory. Despite the limitations of the study, the results indicate that HRAs can be primarily due to the need for nutritional support. On the contrary, cancer pain phenomena such as BTcP, do not seem to affect unexpected HRAs, although when BMI ≥ 25 kg/m^2^, the non-predictable BTcP is more predictive for HRAs. These conclusions are the result of an analysis of a non-representative subsample and further prospective clinical studies should be conducted to verify these findings. The aim is the planning of calibrated programs for cancer patients at higher risk of hospitalization.

## Figures and Tables

**Table 1:** Data collection and variables.

Data Collected	Variable(s)
**Demographic information**	Age Gender
**Anthropometric data**	Weight Height BMI
**Clinical data**	Type of primary tumor Surgical resection Metastases Bone metastases Cancer stage Line of chemotherapy (first or subsequent lines) ECOG-PS Radiotherapy treatment
**Biochemical parameters**	Serum albumin ^ ESR Leukocytes/neutrophils ratio
**Nutritional support prescription**	Enteral/parenteral nutrition
**Pain information**	Type of background pain BTcP features *
**Analgesic therapy**	Type and dosage of opioids for background pain and BTcP
**HRAs**	Number

Abbreviations: BMI, body mass index; ECOG-PS, Eastern Cooperative Oncology Group performance status; ESR, erythrocyte sedimentation rate; BTcP, breakthrough cancer pain; HRA, hospital readmission. ^ cut-off 3.5 g/dL. * Predictable or not; number of weekly episodes; intensity; type; and site.

**Table 2:** Patients’ features (*n* = 96).

Features	*n (%)*
**Age**	
Mean (SD)	70 (11)
Median (IQR)	72 (61, 79)
**Gender**	
Female	48 (50%)
Male	48 (50%)
**BMI**	
Mean (SD)	24.8 (4.1)
Median (IQR)	25.0 (22.0, 27.7)
<25 kg/m^2^	52 (54%)
≥25 kg/m^2^	44 (56%)
**Cancer type**	
Esophageal or gastric	17 (18%)
Colon-rectum	51 (53%)
Pancreas,	28 (29%)
Gallbladder, biliary tract	
**Bone Metastasis**	
Yes	15 (16%)
No	81 (84%)
**Serum Albumin**	
≤3.5 g/dL	49 (51%)
>3.5 g/dL	47 (49%)
**Nutritional Support**	
Yes	18 (19%)
No	78 (81%)
**BTcP**	
Not predictable	71 (74%)
Predictable	25 (26%)
**Type of BTcP**	
Nociceptive	51 (53%)
Neuropathic or both	45 (47%)
**Radiotherapy**	
Yes	11 (11%)
No	85 (89%)
**HRA**	
≤10	24 (25%)
11–22	38 (40%)
<22	34 (35%)

Abbreviation: BMI, body mass index; BTcP, breakthrough cancer pain; HRA, hospital readmission.

**Table 3:** Association analysis.

	BMI	CANCER	MTX	ALB	NUTR	BTcP	RADIO	HRA
BMI	-	-	-	-	-	-	-	-
CANCER	0	-	-	-	-	-	-	-
MTX	0	1	-	-	-	-	-	-
ALB	0	0	0	-	-	-	-	-
NUTR	0	1	1	1	-	-	-	-
BTcP	1	0	1	0	0	-	-	-
RADIO	0	0	1	0	1	0	-	-
HRA	0	1	0	0	1	0	0	-

Abbreviations: BMI, body mass index; MTX, metastasis; ALB, albumin; NUTR, nutritional support; BTcP, breakthrough cancer pain; RADIO, radiotherapy; HRA, hospital readmission. Notes: 0 = no significant association; 1 = significant association (Pearson’s Chi-square test, 95% significance).

**Table 4:** Main indicators of the proposed models.

	Knowledge-Based	BIC-Based
Directed arches	11	8
Average Markov-Blanket size	4.50	2.25
Average neighborhood size	2.75	1.75
Average branching factor	1.38	0.88
Penalization coefficient	-	2.28
Step of the learning procedure	-	54

## Data Availability

The data used for the Bayesian network analysis are available at Cascella M., Racca E. Dataset_Racca, Zenodo.org 2022. Doi: 10.5281/zenodo.6769798.

## Abbreviations

HRAs, hospital readmissions; GI, gastrointestinal; HRQoL, health-related quality of life; IOPS-MS, Italian Oncologic Pain Multicentric Survey; BTcP, breakthrough cancer pain; BMI, body mass index; ECOG-PS, Eastern Cooperative Oncology Group performance status; MTX, metastasis; ALB, albumin; AIC, Akaike Information Criteria; BIC, Bayesian Information Criteria; DAG, directed acyclic graphs.
